# FDG PET/CT for assessing tumour response to immunotherapy: Report on the EANM symposium on immune modulation and recent review of the literature

**DOI:** 10.1007/s00259-018-4171-4

**Published:** 2018-10-05

**Authors:** Nicolas Aide, Rodney J. Hicks, Christophe Le Tourneau, Stéphanie Lheureux, Stefano Fanti, Egesta Lopci

**Affiliations:** 1 Nuclear Medicine Department, Caen University Hospital, Caen, France; 2 Normandie University, Caen, France; 3 INSERM 1086 ANTICIPE, Normandie University, Caen, France; 4 EANM Oncology Committee, Vienna, Austria; 5 Centre for Molecular Imaging, Department of Cancer Imaging, Peter MacCallum Cancer, Melbourne, Australia; 6 Sir Peter MacCallum Department of Oncology, University of Melbourne, Melbourne, Australia; 7 Department of Medical Oncology, Institut Curie, Paris & Saint-Cloud, France; 8 INSERM U900 Research Unit, Saint-Cloud, France; 9 Princess Margaret Cancer Centre, Department of Medical Oncology, University of Toronto, Toronto, ON Canada; 10 Nuclear Medicine, Policlinico S. Orsola, Università di Bologna, Bologna, Italy; 11 Nuclear Medicine Department, Humanitas Clinical and Research Hospital, Rozzano, Milan Italy

**Keywords:** Immunotherapy, Immune checkpoint inhibitors, Pseudoprogression, Hyperprogression, Immune-related side effects, Therapy response

## Abstract

This paper follows the immunotherapy symposium held during the European Association of Nuclear Medicine (EANM) 2017 Annual Congress. The biological basis of the immune checkpoint inhibitors and the drugs most frequently used for the treatment of solid tumours are reviewed. The issues of pseudoprogression (frequency, timeline), hyperprogression and immune-related side effects are discussed, as well as their implications for patient management. A review of the recent literature on the use of FDG PET for assessment of immunotherapy is presented, and recommendations are provided for assessing tumour response and reporting immune-related side effects with FDG PET based on published data and experts’ experience. Representative clinical cases are also discussed.

## Background

### Immune checkpoints and available inhibitors

Beside surgery, radiation, chemotherapy and molecularly targeted therapy, immunotherapy has recently emerged as an important advance in cancer treatment. Immunotherapy radically differs from other strategies in relying on the reactivation of the immune system to recognize and kill cancer cells [1]. This strategy is based on evidence that development of cancer is enabled by dysregulation and exploitation of otherwise physiological pathways [2]. The use of immunomodulatory monoclonal antibodies that directly enhance the function of components of the antitumour immune response, such as T cells, or block immunological checkpoints that would otherwise restrain effective antitumour immunity has recently been actively investigated in oncology.

To date, the main immunotherapeutic approach that has been translated into survival benefit and is currently used in practice is the blockade of immune checkpoints. Broadly, the two most effective classes of agent are directed, alone or in combination, towards cytotoxic T lymphocyte-associated protein 4 (CTLA-4) or the programmed cell death protein 1 (PD1) or the PD1/programmed cell death protein ligand 1 (PD1/PD-L1) axis, which are negative regulators of T cell immune function. CTLA-4 is recruited to the surface of regulatory T cells, and interacts with B7 receptors found on antigen-presenting cells, which results in the downregulation of any further T cell activation and immune response expansion [3]. Therefore, CTLA-4 is induced in T cells at the time of initial response to antigen and regulates the amplitude of the early stages of T cell activation [4]. PD1, a well-studied immune checkpoint molecule, is a transmembrane glycoprotein expressed on a variety of immune cells [5]. PD-L1 and PD-L2, the ligands for PD1, have distinct patterns of expression and can be induced, or constitutively expressed, on an array of cells including various tumour cells. PD-L1 is expressed on leucocytes, on nonhaematopoietic cells and in nonlymphoid tissues, and can be induced on parenchymal cells by inflammatory cytokines (IFN-γ) or tumorigenic signalling pathways. In normal settings, PD-L1 is expressed at low levels. However, many cancers show increased levels of expression of the molecule.

PD-L2 is primarily expressed on dendritic cells and monocytes but can be induced on a wide variety of other immune cells and nonimmune cells, depending on the local microenvironment. When engaged by one of its ligands, PD1 inhibits kinases that are involved in T cell activation. As PD1/PD-L1 binding inhibits T cell receptor-mediated positive signalling, the major role of the PD1 pathway is not at the initial T cell activation stage but rather in regulating cytotoxic responses in tissues by effector T cells recognizing antigen in peripheral tissues [4]. PD1 has a higher binding affinity for PD-L2 than for PD-L1, and this difference may be responsible for differential contributions of these ligands to immune responses [6]. This biological dysregulation of CTLA-4 and PD1/PD-L1 expression is suspected to play a key role in tumour immune evasion and has become an attractive target for therapeutic intervention. CTLA-4 blockade allows activation and proliferation of more T cell clones and reduces Treg-mediated immunosuppression. PD1/PD-L1 pathway blockade restores the activity of antitumour T cells that have become quiescent.

The CTLA-4 inhibitor, ipilimumab, has been shown to improve survival rates in melanoma patients. PD1/PD-L1 inhibitors (of which the first validated agents were pembrolizumab and nivolumab) have been shown to improve survival rates among patients with various tumour types, including melanoma, lung, head and neck, and bladder cancers. Typically, these drugs are given intravenously every 2 to 3 weeks and have been shown to produce a durable complete response (CR) in a variable but small proportion of patients. Patients whose tumours or immune cells express PD-L1 have a higher likelihood of benefiting from treatment with PD1/PD-L1 inhibitors, although PD-L1-negative patients have also been shown to respond.

Since not all patients respond to single-agent immunotherapy, hundreds of combination trials are ongoing. Different combination strategies are under investigation including with standard chemotherapy, targeted agents and antiangiogenic agents. Combinations also include other immunotherapeutic agents, such as LAG3 inhibitors and OX40 agonists. In the locally advanced setting, treatment with combinations of PD1/PD-L1 inhibitors has been shown to be feasible in patients with various tumour types. Since radiation induces the release of tumour antigens, also known as neoantigens, there is strong rationale supporting the use combinations of either external beam or radionuclide therapy and immune checkpoint inhibitors [7].

### Immune-related side effects

By reactivating the immune system, these immunotherapies have led to the development of new toxicity profiles, also called immune-related adverse events (irAE). IrAEs can involve many organ systems, and their management is radically different from that of adverse events from cytotoxic drugs [8]. There is a wide variety of irAEs, with the endocrine, cutaneous and gastrointestinal systems being the most commonly affected (for example, thyroiditis, rash and gastrointestinal irAEs, respectively). Pneumonitis, arthritis and myalgia have also been reported. The irAE pattern is different across immune checkpoint inhibitor classes and could be driven by the different patterns of immune cell activation that can occur with different classes of immune therapy [9]. The rapid identification of these irAEs and the initiation of systemic immunosuppression, for example with corticoids [10, 11], can improve patient outcomes.

### Pseudoprogression and hyperprogression

Patterns of response to immunotherapeutic agents also differ from those to chemotherapeutic and molecularly targeted agents. First, although responses usually occur early, they can also be delayed. Second, responses may be preceded by apparent disease progression, retrospectively termed pseudoprogression. These patterns of response have mainly been reported in melanoma patients receiving anti-CLTA4 agents, with approximately 15% of patients experiencing pseudoprogression [12]. Pseudoprogression appears to be much rarer in all other tumour types (less than 3%), especially with the use of anti-PD1/PD-L1 agents, indicating that in the vast majority of patients progression seen on morphological imaging is authentic progression. Pseudoprogression should only be considered when the clinical condition of the patient is concomitantly improving. Patients whose clinical condition is not improving and who have disease progression on imaging should discontinue immunotherapy. The risk of continuing treatment beyond progression is that it may prevent commencement of a new line of treatment once the progression is confirmed because of clinical deterioration.

**Fig. 1:**
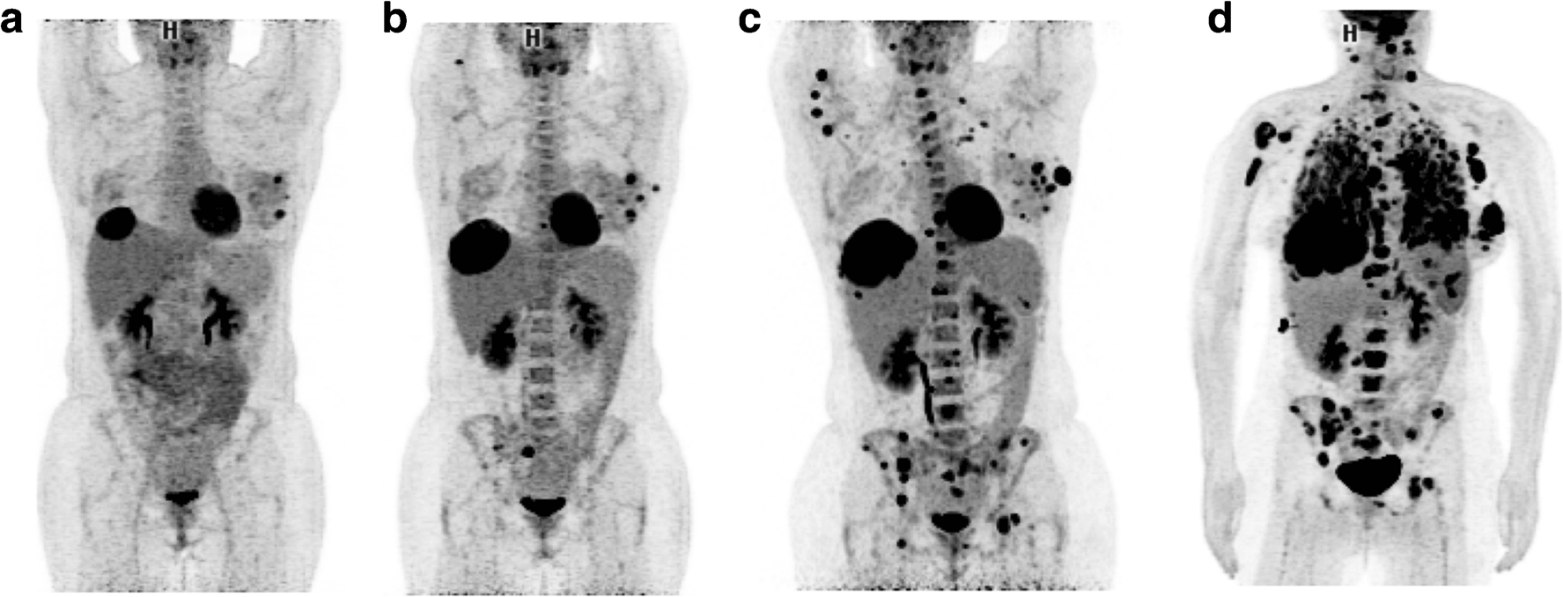
FDG PET images in a melanoma patient with breast and liver metastases treated with nivolumab after progression under anti-BRAF and anti-MEK treatment. **a** Baseline scan. **b** Early scan after two cycles shows progression in the breast and liver lesions as well as the appearance of bone metastases. **c** Scan after six cycles confirms the findings of progression. The case was classified as hyperprogression during immunotherapy (**d**)

**Fig. 2:**
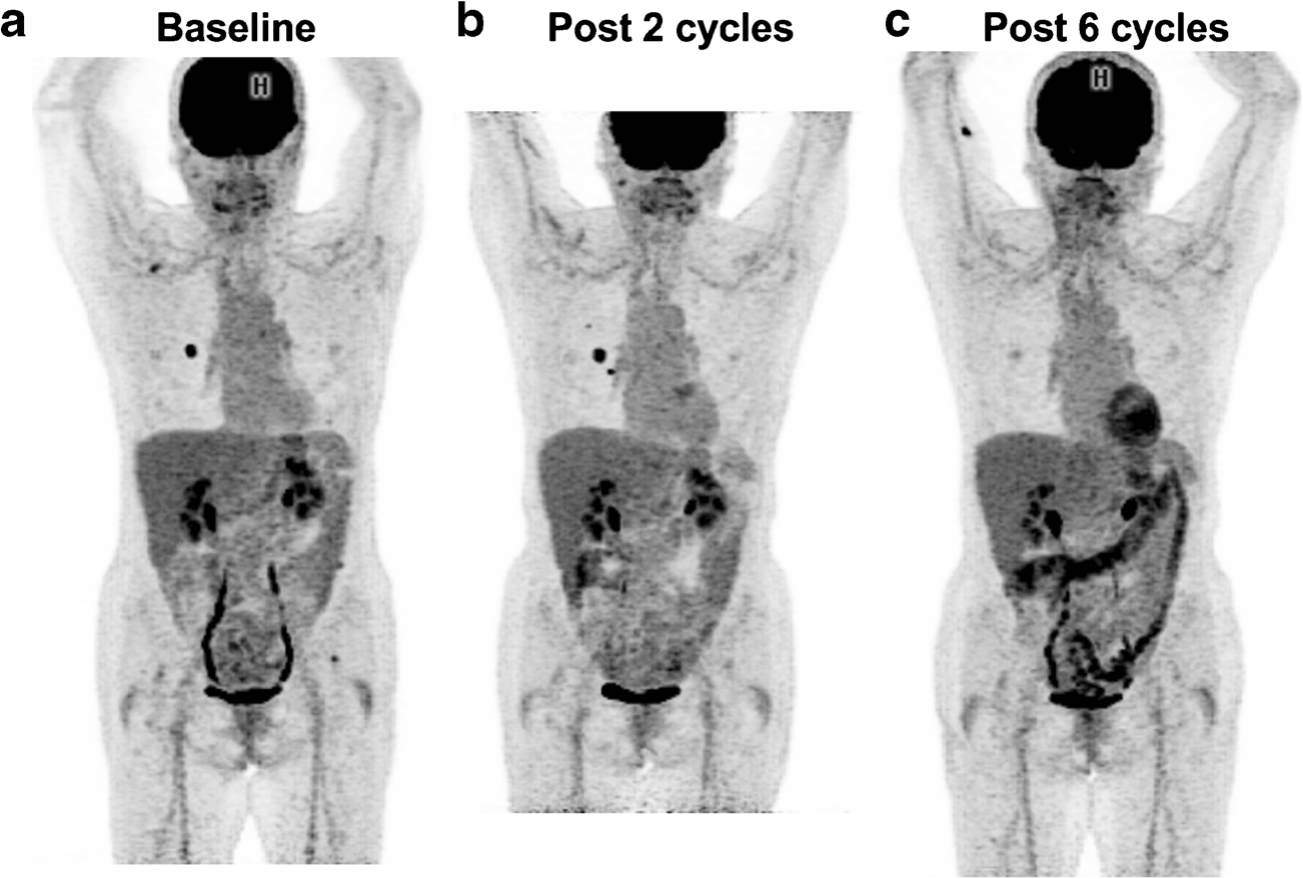
FDG PET images in a melanoma patient with lung metastases treated with nivolumab. **a** Baseline scan. **b** Early scan after two cycles shows two new lung lesions. **c** Scan after six cycles shows a complete metabolic response. Note the appearance of diffuse colonic uptake reported as possible colitis. The patient had no digestive symptoms. The progression seen after two cycles was considered to represent pseudoprogression

We and others have reported cases of hyperprogression, which is defined as an acceleration of tumour growth kinetics [13, 14]. Some positive pivotal phase III trials have shown worse overall survival in patients receiving immune checkpoint inhibitors than in control patients during the first few months, supporting the concept of hyperprogression [15, 16]. Retrospective studies have shown that a substantial proportion of patients show an increase in their tumour volume or sum of the largest diameters by more than 100% over time on immunotherapy, as compared to their previous treatment. Although these studies had no control arm, they suggested that immunotherapy might be detrimental in some cancer patients [13, 14, 17]. While it is essential to seek robust biomarkers of hyperprogression, it is important that clinicians interrupt treatment early if hyperprogression is suspected. Figures 1 and 2 illustrate cases of hyperprogression and pseudoprogression identified on FDG PET.

### Current methods for response assessment with CT (from irRECIST to iRECIST)

Modification of the existing definitions used for defining therapeutic response based on morphological imaging techniques using the Response Evaluation Criteria in Solid Tumours (RECIST) was largely driven by the observation of pseudoprogression in a subgroup of patients treated primarily with ipilumumab. There are currently two slightly different modifications, known as irRECIST and iRECIST. The latter was developed by the RECIST Working Group and therefore is the version more likely to be adopted widely [18]. Essentially, iRECIST has a new category of unconfirmed progression (iUPD) that requires progression to be confirmed by a further follow-up scan. This can also include identification of new lesions, which need to be categorized as measurable or not using RECIST 1.1 principles but that are not included in the sum of target lesions measured at baseline assessment. These guidelines further suggest that if the patient is clinically stable, treatment should be continued.

## Review of the use of FDG PET in immunotherapy response assessment

### Evolution of metabolic or combined response criteria

The first PET-based response criteria embedding metabolic response to treatment in solid tumours were proposed by the European Organisation for Research and Treatment of Cancer (EORTC) in 1999 [19]. Based on these criteria, a complete metabolic response (CMR) is reached when all tumour lesions are no longer detectable against adjacent background activity, whereas progressive metabolic disease (PMD) is defined as an increase in SUVmax of ≥25% from baseline imaging or the appearance of new metastatic lesions. The EORTC criteria do not specify the number of lesions to be measured or the minimum measurable lesion SUVmax, but rather refer to the background activity for the definition of CMR. A partial metabolic response (PMR) is defined as a reduction in SUVmax of between 15% and 25% or >25% after one or more cycles of chemotherapy. Stable metabolic disease (SMD) is considered a response not classifiable in any of the other categories. The EORTC criteria were the first to be applied for the assessment of response of solid tumours to immunotherapy [20]. A summary of the available and/or proposed response criteria with for use with FDG PET is presented in Table 1.

**Table 1:** Available and/or proposed response criteria for use with FDG PET

Response	EORTC^a^	PERCIST^b^	PECRIT^c^			PERCIMT^d^	
Complete response (CR)	Complete resolution of FDG uptake	Disappearance of all metabolically active tumours	RECIST 1.1 (disappearance of all target lesions; reduction in short axis of target lymph nodes to <1 cm; no new lesions)		Clinical benefit	Complete resolution of all preexisting ^18^F-FDG-avid lesions; no new ^18^F-FDG-avid lesions	Clinical benefit
Partial response (PR)	Minimum reduction of ±15–25% in tumour SUV after one cycle of chemotherapy, and >25% after more than one treatment cycle	Decline in SULpeak by 0.8 unit (>30%) between the most intense lesion before treatment and the most intense lesion after treatment	RECIST 1.1 (decrease in target lesion diameter sum >30%)		Clinical benefit	Complete resolution of some preexisting ^18^F-FDG-avid lesions. No new, ^18^F-FDG avid lesions.	Clinical benefit
Stable disease (SD)	increase in SUV of less than 25% or a decrease of less than 15%	Does not meet other criteria	Does not meet other criteria	Change in SULpeak of the hottest lesion of >15%	Clinical benefit	Neither PD nor PR/CR	Clinical benefit
				Change in SULpeak of the hottest lesion of ≤15%	No clinical benefit		
Progressive disease (PD)	Increase in tumour FDG uptake of >25%; increase in maximum tumour of >20%; new metastases	Increase in SULpeak of >30% or the appearance of a new metabolically active lesion	RECIST 1.1 (increase in target lesion diameter sum of >20% and at least 5 mm or new lesions)		No clinical benefit	Four or more new lesions of <1 cm in functional diameter or three or more new lesions of >1.0 cm in functional diameter or two or more new lesions of more than 1.5 cm in functional diameter	No clinical benefit

*EORTC* European Organisation for Research and Treatment of Cancer*, PERCIST* PET Response Criteria in Solid Tumors, *PECRIT* PET/CT Criteria for Early Prediction of Response to Immune Checkpoint Inhibitor Therapy (combined RECIST 1.1 and PERCIST), *PERCIMT* PET Response Evaluation Criteria for Immunotherapy, *SUV* standardized uptake value, *SUL* SUV normalized by lean body mass

^a^*Measurable lesions*: the most FDG-avid lesions in terms of SUVs normalized by body surface area. *New lesions*: as progressive disease. *Number of lesions*: not specified

^b^*Measurable lesions*: minimum tumour SUL 1.5 times the mean SUL of the liver. *New lesions*: as progressive disease. *Number of lesions*: changes in the sum of up to five lesions as secondary measure to assess response

^c^*Measurable lesions*: RECIST 1.1 (1 cm on CT; longest diameter, except in lymph nodes); PERCIST (minimum tumour SUL 1.5 times the mean SUL of the liver). *New lesions*: as progressive disease. *Number of lesions*: RECIST 1.1 (up to five, maximum two per organ); PERCIST (changes in the sum of up to five lesions as secondary measure to assess response)

^d^*Measurable lesions*: FDG-avid lesions considered with regard to their absolute number and functional size (>1.0 cm or >1.5 cm) measured in centimetres on the fused PET/CT images. *New lesions*: as progressive disease, based on number and functional diameter. *Number of lesions*: up to five target lesions per patient before and after treatment

In 2009, ten years after the introduction of the first PET-based criteria, Wahl et al. proposed the PET Response Criteria in Solid Tumors (PERCIST) [21]. They are rather similar to the EORTC criteria, and therefore the response assessment provided by PERCIST tend to give very similar results, but with some differences in terms of response classification (Table 1). The major innovations of PERCIST were the use of SUV lean (SUV normalized by lean body mass, or SUL) for the assessment of tumour response and the identification of a minimum tumour SUL equivalent to 1.5 times the mean SUL of the liver for a lesion to be evaluable. PERCIST also show some similarities with the morphological criteria (i.e. RECIST), by recommending the measurement of SUL in up to five tumours (up to two per organ) corresponding to the target lesions. These criteria were also the first to introduce the concept of SULpeak within the area of highest uptake in the tumour, which can be measured within a spherical region of interest of diameter 1.2 cm (1 cm^3^ volume). The use of PERCIST criteria with respect to response to immunotherapy has been described only rather recently [22].

In an attempt to find the perfect fit between morphological and metabolic responses, Cho et al. [23] evaluated different criteria (i.e. RECIST 1.1, irRC, PERCIST and EORTC) in a small cohort of 20 patients with advanced melanoma treated with either ipilimumab (*n* = 17) or nivolumab (*n* = 3). This imbalance in type of treatment agent somewhat limits the generalizability of these criteria since the authors found pseudoprogression, which is implied by these criteria, mainly with ipilumumab and seldom with anti-PD1/PD-L1 agents. Notwithstanding this observation, the cohort was prospectively investigated after days 21–18 and 4 months following the start of therapy with the aim of defining the best combination for response assessment in immunotherapy. In particular, the best combination of parameters, which were termed by the authors as PECRIT (PET/CT Criteria for Early Prediction of Response to Immune Checkpoint Inhibitor Therapy), included either a change in the sum of RECIST 1.1-based target lesion diameters (method 1), and a change in SULpeak of >15.5% of the hottest lesion (method 3) [23]. Combining morphological and metabolic criteria led to an accuracy of 95% (sensitivity 100%, specificity 93%). One of the most interesting aspects of this study is the introduction of clinical benefit (CB) into the definition of response (Table 2). In particular, this classification applies to patients with a complete response (CR) or partial response (PR) according to morphological criteria plus all patients with stable disease (SD) with a decrease in SULpeak greater than the cut-off value of 15.5%.

**Table 2:** Principal studies investigating the role of FDG PET/CT in the evaluation of response of solid tumours to immunotherapy

Reference	Study type	Number of patients	Tumour	Treatment	Response criteria	Results
[20]	Prospective	22	Melanoma	Ipilimumab	EORTC after two cycles of treatment (early) and at the end of treatment after four cycles (late)	Early response evaluation after (two cycles) is predictive of final treatment outcome in patients with PMD and SMD
[26]	Prospective	27	Melanoma	20 pembrolizumab, 7 nivolumab	Visual analysis (qualitative visual inspection, positive when FDG uptake greater than background activity or hepatic uptake; Deauville score)	43% of patients who had residual disease by CT criteria, either PR or SD, were FDG-negative
[36]	Prospective	31	Melanoma	Ipilimumab	Fractal and multifractal analysis before and after two and after four cycles of treatment	Operator-independent method with a correct classification rate of 83.3%
[23]	Prospective	20	Melanoma	16 Ipilimumab, 1 nivolumab, 3 BMS-936559	RECIST 1.1 and PERCIST at early (4 weeks) and late assessment (4 months)	Combined anatomical and functional data at 21–28 days (PECRIT) criteria predicted response with 100% sensitivity, 93% specificity and 95% accuracy. Introduction of clinical benefit in response criteria
[22]	Prospective	24	NSCLC	Nivolumab	RECIST 1.1 versus PERCIST; additional semiquantitative analyses (SUVmax, MTV, TLG)	Metabolic response on PET (especially TLG) associated with therapeutic response and survival at 1 month after nivolumab
[28]	Prospective	27	NSCLC	23 nivolumab, 4 pembrolizumab	Baseline semiquantitative analysis	SUVmax ≤17.1 (sensitivity 88.9%) or a SUVmean ≤8.3 (sensitivity 100%) identified fast progression after 8 weeks of therapy
[24]	Prospective enrolment, retrospective PET analysis	41	Melanoma	Ipilimumab	RECIST and appearance of new FDG-avid lesions (PERCIMT); patients were dichotomized into those with and those without clinical benefit	A cut-off of four newly emerged FDG-avid lesions on posttreatment PET/CT gave reliable indication of treatment failure
[25]	Prospective	41	Melanoma	Ipilimumab	EORTC and PERCIMT after two cycles of immunotherapy	PERCIMT to interim PET/CT provides a more sensitive predictor of final response than EORTC criteria

*EORTC* European Organisation for Research and Treatment of Cancer, *RECIST* Response Evaluation Criteria In Solid Tumors, *PERCIST* PET Response Criteria in Solid Tumors, *PECRIT* PET/CT Criteria for Early Prediction of Response to Immune Checkpoint Inhibitor Therapy (combined RECIST 1.1 and PERCIST), *PERCIMT* PET Response Evaluation Criteria for Immunotherapy, *NSCLC* non-small cell lung cancer, *MTV* metabolic tumour volume, *TLG* total lesion glycolysis, *PMD* progressive metabolic disease, *SMD* stable metabolic disease, *PR* partial response, *SD* stable disease

CB also appears to be the main goal for the criteria recently proposed by the group from Heidelberg for FDG PET evaluation of response to immunotherapy in patients with melanoma [24, 25]. The PERCIMT (PET Response Evaluation Criteria for Immunotherapy) classification takes into consideration the observed relevance of the absolute number of new lesions on FDG PET scan and its more robust predictive role compared to pure SUV changes during the course of treatment with ipilimumab. In particular, the authors dichotomized patients according to CB from the treatment (CR/PR and SD) or no CB from the treatment, i.e. progressive disease determined as the appearance of: (a) four or more new lesions <1 cm in functional diameter, (b) three or more new lesions >1.0 cm in functional diameter, or ©) two or more new lesions >1.5 cm in functional diameter. In all cases, the functional diameter is considered the lesion diameter measured in centimetres based on the fused PET/CT images.

In a cohort of 20 patients with advanced melanoma treated with either ipilimumab or nivolumab [23] with response to treatment assessed early (days 21–18) and late (4 months) after the start of therapy, separate early assessment with PERCIST and EORTC criteria demonstrated suboptimal accuracies of 70% and 65%, respectively, for the prediction of best overall response at 4 months. Therefore, the authors proposed PECRIT (the combined criteria) that had an accuracy of 95%, as being better associated with CB in melanoma patients treated with immunotherapy.

### FDG PET for immunotherapy response assessment

The principal studies investigating the role of FDG PET/CT in the evaluation of response of solid tumours to immunotherapy are summarized in Table 2. Given the major impact that immunotherapy with checkpoint inhibitors has had on the treatment of metastatic melanoma, it is not surprising that the first report on metabolic response during the course of immunotherapy related to FDG PET evaluation after two cycles of ipilimumab and at the end of treatment in 22 patients with melanoma [20]. In this initial analysis, the EORTC criteria were used for response assessment and showed that early PET (after two cycles) was predictive of outcome (late response) in patients with PMD and SMD. Already in this first report, the authors recognized the appearance of new lesions, conventionally defining disease progression, as being a potential cause of response misclassification.

One year later, another group [26] investigated the role of residual metabolic activity on FDG PET in patients with metastatic melanoma presenting with a prolonged response to immunotherapy with anti-PD1 agents (i.e. pembrolizumab and nivolumab). No defined metabolic response criteria were used for the definition of response on PET, and the authors relied mostly on visual assessment and qualitative analysis based on background tissue comparisons in a manner similar to the Deauville score. Overall, 27 patients were analysed, of whom 15 (56%) had a positive FDG PET scan with a biopsy-proven melanoma residue in eight (62%). Of the remaining 12 patients with a negative PET scan, six presented with a residual lesion on CT and five had ceased treatment, but none of these patients showed recurrence during 6–10 months of follow up. In summary, 43% of patients with residual disease based on CT criteria (either PR or SD) were negative on FDG PET. Occasionally, metabolically active lesions in patients with CB from immunotherapy in the long term may show positive findings on PET that can be considered to be a result of immune cell infiltrates rather than melanoma localizations.

A more recent study including 20 patients with advanced melanoma treated with either ipilimumab or nivolumab [23] assessed responses to treatment early (days 21–18) and late (4 months) after starting therapy. Various morphological criteria (RECIST 1.1, irRC) and metabolic criteria (PERCIST and EORTC) were directly compared to define the best combination for the assessment of response to immunotherapy with checkpoint inhibitors. Interestingly, the authors found low inter-criteria agreement (kappa = 0.48–0.7) among RECIST, PERCIST and EORTC in the early assessment, whereas there was good to excellent agreement between CT modalities and PET in the late evaluation.

Another recent study prospectively enrolled 41 patients with metastatic melanoma treated with ipilimumab [24] and evaluated at baseline and 3 months later. Changes in SUVmax and SUVmean during the course of immunotherapy were not correlated with clinical response (*t* test; *p* = 0.06 and 0.05, respectively), whereas the number of new lesions was able to define disease progression (Tables 1 and 2), improving the identification of patients who will show CB (Wilcoxon test, *p* < 0.0001). Moreover, optimal cut-off points for the total number of new lesions on the basis of their functional diameter (measured diameter on fused PET/CT images) were defined: (a) four new lesions led to an observed sensitivity of 84% and a specificity of 100%, (b) a functional size >1.0 cm for a cut-off of three new lesions led to a sensitivity of 90% and a specificity of 90%, and ©) a functional size >1.5 cm for a cut-off of two new lesions led to a sensitivity of 94% and a specificity of 90%. By combining all the available data, the authors proposed the PERCIMT criteria.

The group from Heidelberg in the same cohort of patients also investigated the applicability of PERCIMT at interim evaluation after two cycles of immunotherapy (Table 2) and compared the results with those using the EORTC criteria [25]. Patients were divided into two groups, those showing metabolic benefit, including SMD, PMR and CMR, and those showing no metabolic benefit (PMD). Overall, agreement between the two sets of response criteria was poor (kappa = 0.46; McNemar test *p* = 0.001). The PERCIMT showed a significantly higher sensitivity than the EORTC criteria in predicting CB (93.6% versus 64.5%, respectively; *p* = 0.004), but did not show a significantly higher specificity (70.0% versus 90.0%, respectively; *p* = 0.5) in predicting no CB. The superiority of the new proposed response criteria is therefore questionable, first because of the limited number of patients on which the PERCIMT were developed (*n* = 41), and second because the EORTC criteria appear to be better at identifying patients who will not respond to ipilimumab than the PERCIMT, although the difference in specificity did not reach significance.

Only a few studies have investigated the response of tumour types other than melanoma to immunotherapy, especially non-small cell lung cancer (NSCLC), and some are case reports [22, 27–29]. In a recent study assessing response of NSCLC to immunotherapy [22], 24 patients treated with nivolumab were investigated at baseline and 1 month after the start of treatment. Response was determined using either morphological (RECIST 1.1) or PERCIST criteria, along with SUVmax, metabolic tumour volume (MTV) and total lesion glycolysis (TLG). The value of PET in predicting PR and progressive disease was significantly higher than that of CT. This was also shown in a multivariate analysis that confirmed FDG uptake (i.e. TLG) after administration of nivolumab as an independent factor predicting PFS (HR 3.624; *p* < 0.001) and OS (HR 2.461; *p* = 0.012).

In a recent study of the use of an anti-PD-L1 agent, atezolizumab, in the treatment of NSCLC, the potential of FDG PET/CT for assessing response was evaluated. FDG PET scans at baseline and 6 weeks were evaluable in 103 patients. Patients with an early FDG response at 6 weeks according to the EORTC criteria achieved a higher objective response rate on subsequent CT than metabolic nonresponders (17/ 23, 73.9% versus 5/80, 6.3%). Possible pseudoprogression was identified in only two patients [30].

### Additional considerations with respect to tumour metabolism

Another important aspect to be considered, particularly during baseline evaluation, is that FDG PET can provide useful information on the metabolic state of the tumour microenvironment and on the expression of checkpoint inhibitors. Indeed, in patients with NSCLC, there is a statistically significant association between tumour metabolic parameters on PET and PD1/PD-L1 expression, along with the presence of CD8+ tumour infiltrating lymphocytes (TILs), in resected tumour specimens [31, 32]. The presence of immune infiltrate is already known as a good predictor of response to immunotherapy. Several studies have shown that responding patients have a significantly higher expression of CD8+ TILs, and PD1 and PD-L1 cells before treatment than patients with progression [33, 34]. In addition, Mazzaschi et al. [35] found that patients with CB and longer progression-free survival following treatment with nivolumab showed CD8+ lymphocytes with low expression of PD1, while the PD1-to-CD8 ratio was a prognostic factor in univariate and multivariate analyses.

It is not surprising that some initial evidence, although limited to a cohort of 27 patients with NSCLC [28], has shown the value of FDG PET in predicting response to immunotherapy with checkpoint inhibitors. Grizzi et al. [28] found that almost all patients classified as fast progressors after 8 weeks of immunotherapy showed SUVmax ≤17.1 or SUVmean ≤8.3 on baseline PET. The apparently low specificity of these cut-off values, which conversely maintain high sensitivity, is attributable to the fact that response to immunotherapy depends on multiple factors. Imaging and metabolic data, analysed visually, semiquantitatively or with dedicated algorithms [36], are pieces of the “puzzle”. As a consequence, the metabolic characteristics of the tumour and its environment at baseline may be part of a larger panel of predictive factors of response to immunotherapy [37].

## Recommendation on PET scanning and reporting

### PET protocol

**Fig. 3:**
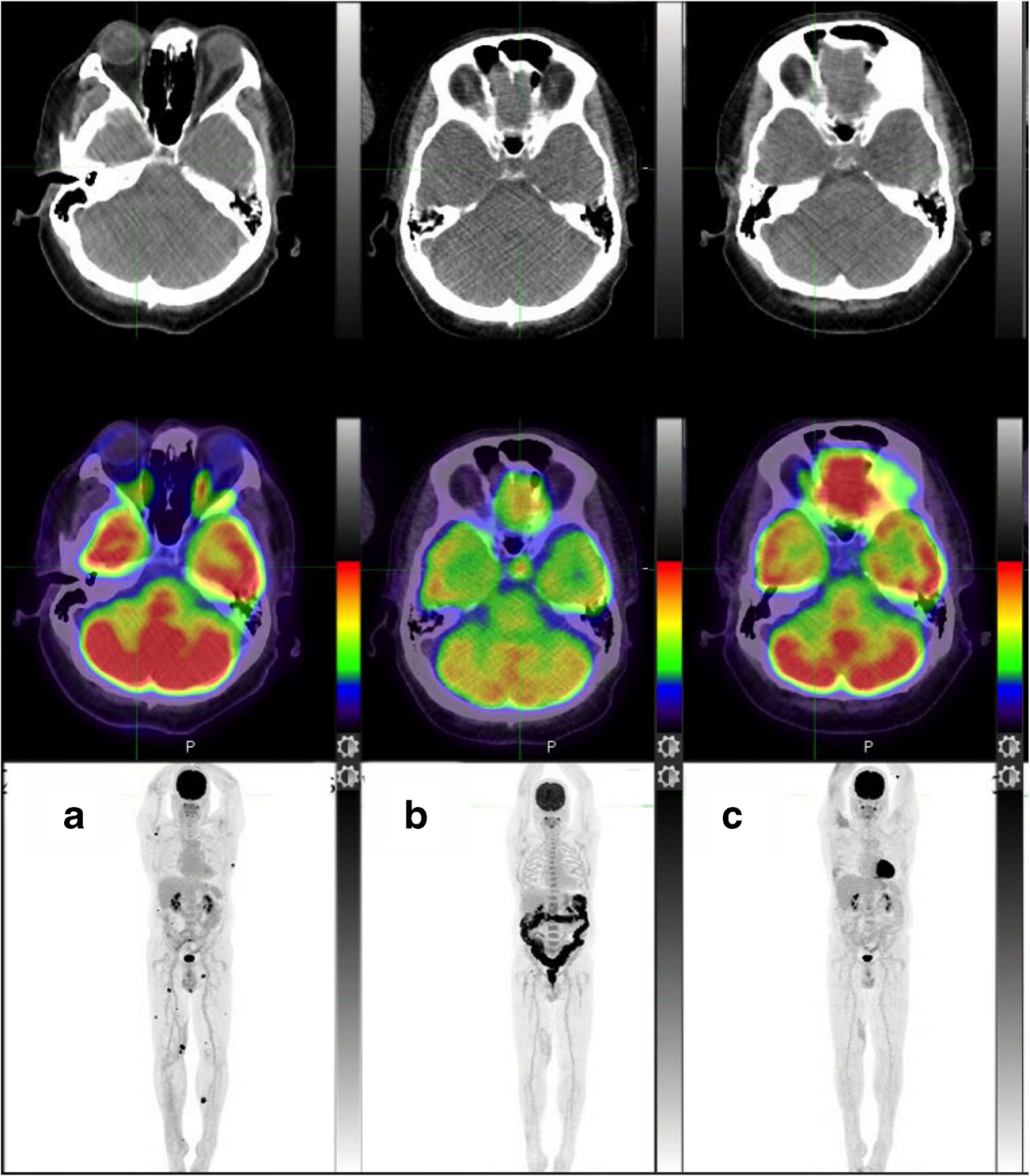
PET/CT imaging in a patient with a previous complete metabolic response of subcutaneous metastases to immunotherapy. **a**, **b** Comparison of the baseline maximum intensity projection image (**a**) with the early posttreatment images (**b**) shows development of increased uptake in the pituitary fossa on the corresponding fused PET/CT image indicating hypophysitis and diffuse colonic uptake indicating colitis, which were confirmed biochemically and clinically. **c** Resolution of both complications is apparent after treatment with corticosteroids

Apart from the usual compliance to PET tumour imaging guidelines, several points regarding the PET acquisition protocol need to be raised. First, if the brain is not systematically included in the field of view, the skull base should be included, so that immune-related side effects involving the pituitary gland are not missed (Fig. 3). Second, in patients with melanoma with a primary located in the lower limbs, a whole-body acquisition is recommended. The number of cycles of immunotherapy since the baseline PET scan and the date of the last infusion should be part of the PET report. A checklist for PET reporting is presented in Table 3.

**Table 3:** Checklist for PET reporting

PET indication		Checklist	
Patient medical examination		Type of immune modulator received (anti-CTLA or anti-PD1 or association in the framework of clinical trials)	
		Number of cycles received and date of the last injection	
		Clinical symptoms congruent with immune-related side effects, with focus on the most severe (colitis and pneumonitis)	
		For diabetic patients, check whether drugs likely to mimic colitis (biguanides) have been withdrawn or not	
Reporting therapy response		Response of target lesion(s)	
		If possible compute and report MATV and TLG	
		If appearance of new lesions:	Report the number of new anatomical sites and the number of new lesions
			If new nodal sites: are they located in the drainage area of the main tumour lesion(s) ?
			In line with the previous item and the next section, check whether new lesions may be related to immune-related side effects (see below) before classifying the patient as PMD
Seeking Immune-related side effects		Keep in mind that they are more common with anti-CTLA (Ipilimumab)	
		Measure the spleen and the liver-to-spleen FDG uptake ratio uptake (inversion?)	
		Consider whether the pattern of new nodal uptake suggests sarcoidosis (lambda sign with or without portocaval nodes, Fig. 6)	
		Refer to baseline scan when an organ frequently showing increased physiological uptake is thought to be involved by an immune-related side effect (thyroid, stomach) (see Fig. 5)	
		Check the pituitary gland (Fig. 3)	
		Any organ may be involved but pay attention to life-threatening adverse effects or those likely to need treatment withdrawal or corticosteroid treatment (colitis and pneumonitis)	
		Bilateral adrenal enlargement and increased uptake is probably due to adrenalitis	
		When immune-related side effects are shown on a previous PET scan, check patient’s recovery (Figs. 3 and 4)	

### PET indication

FDG PET imaging has to be performed before the start of immunotherapy, together with conventional contrast-enhanced CT (ceCT). The metabolic information obtained at this time allows adequate restaging and proper evaluation of disease extent at baseline. The scan should be repeated at the first treatment response evaluation, which in most cancer types is 8 or 9 weeks after the start of immunotherapy, which is generally after two or three cycles of treatment, depending on the regimen used. The added value of FDG PET imaging during treatment is generally found in patients with no morphological response on ceCT or presenting with symptoms, or with signs of irAEs. Along with CB, the presence of a metabolic response despite morphological progression (Table 1) should support clinicians in decision making. Subsequent imaging with FDG-PET is recommended at the end of immunotherapy, before treatment stop.

### Immune-related signs

**Fig. 4:**
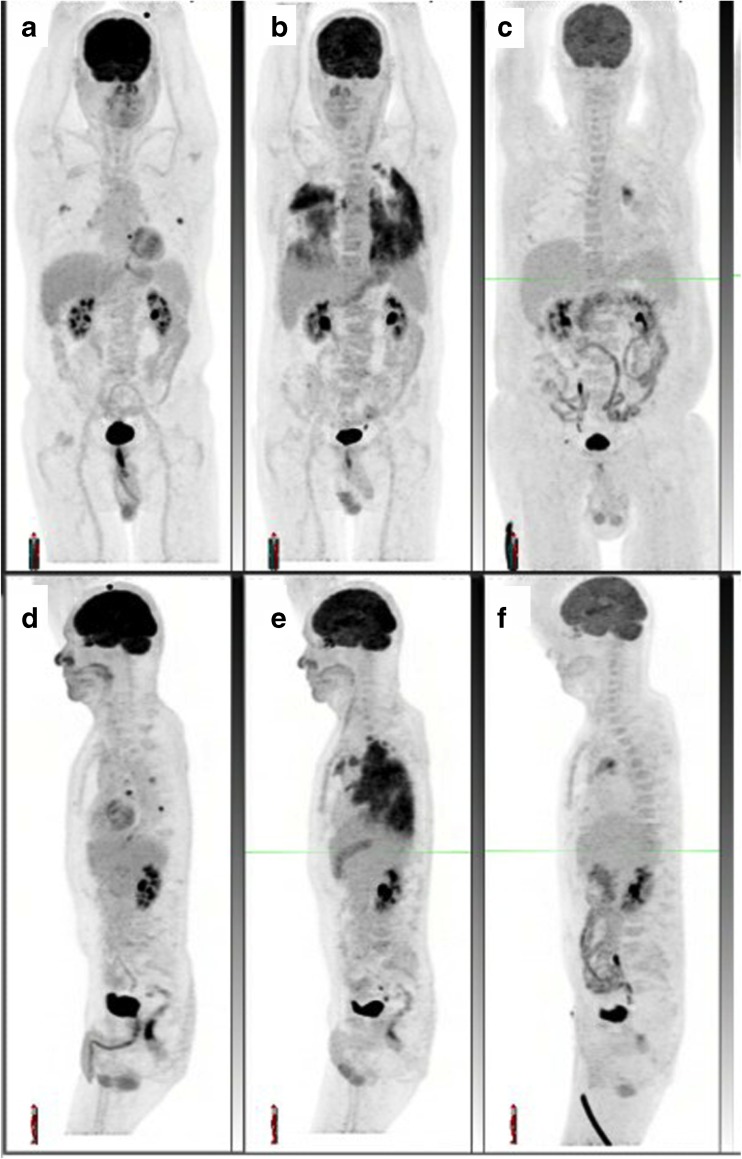
Serial maximum intensity projection images (**a**–**c** anterior, (**d**–**f** ) left lateral) show the development and resolution of pneumonitis. Note the dominance of parenchymal changes in the dependent lung, which is typical. There was a complete metabolic response with low-grade left hilar changes (**c**, **f**) consistent with reactive lymphadenopathy

Response assessment during immunotherapy can be rather challenging since inflammatory reactions can occur during the treatment and are associated with high glucose consumption [37]. This may be associated with pseudoprogression and irAEs and can lead to misinterpretation of FDG PET images. However, FDG PET can show dynamic adaptation of the immune response to checkpoint inhibitors [38, 39]. Moreover, being a whole-body modality, it also allows precise localization of irAEs, which can occasionally become life-threatening; for example, colitis (Fig. 3), pneumonitis (Fig. 4) and pancreatitis. Furthermore, the occurrence of irAEs and the possibility of detecting them on PET may be an additional factor predicting response to immunotherapy, given the evidence that irAEs are associated with the efficacy of PD1 inhibitors in patients with melanoma or NSCLC [40, 41].

Although potentially immune-related inflammatory findings on FDG PET should be reported, these will not necessarily be associated with clinical symptoms (i.e. irAEs). Nevertheless, clinicians should be alert to their presence and should ensure close clinical monitoring since medical intervention may be necessary in selected cases to avoid serious complications. The first sign of immune activity to be checked is spleen enlargement and/or increased uptake leading to an inversion of the liver-to-spleen uptake ratio. Reactive nodes in the drainage basin of the primary tumour may also be seen.

**Fig. 5:**
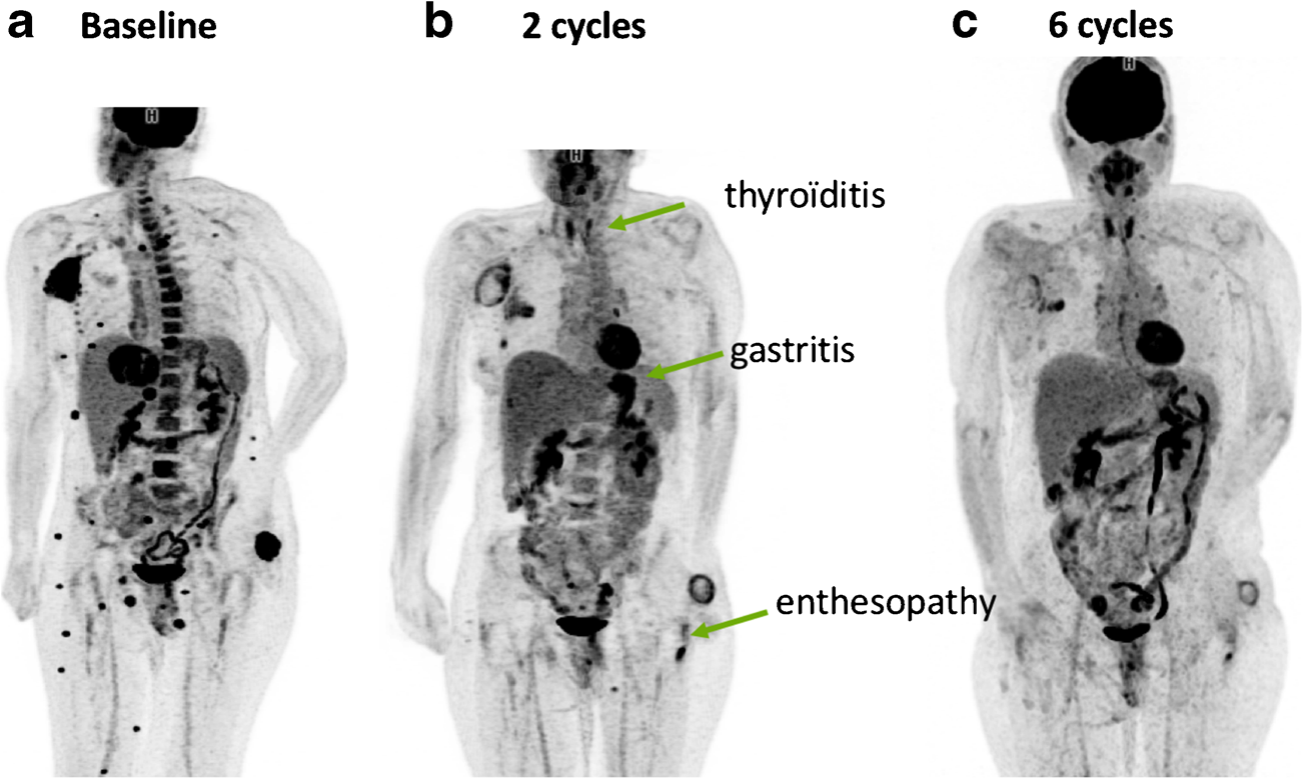
A patient with multiple melanoma metastases (nodes, diffuse bone involvement, multiple soft tissue lesions and solitary liver lesion) receiving nivolumab plus external beam radiation to the right axilla and a soft lesion near the left hip shows an almost complete metabolic response. Multiple signs of immune-related side effects are seen after two cycles of immunotherapy. Note the increased spleen uptake on the baseline scan due to an inflammatory syndrome

**Fig. 6:**
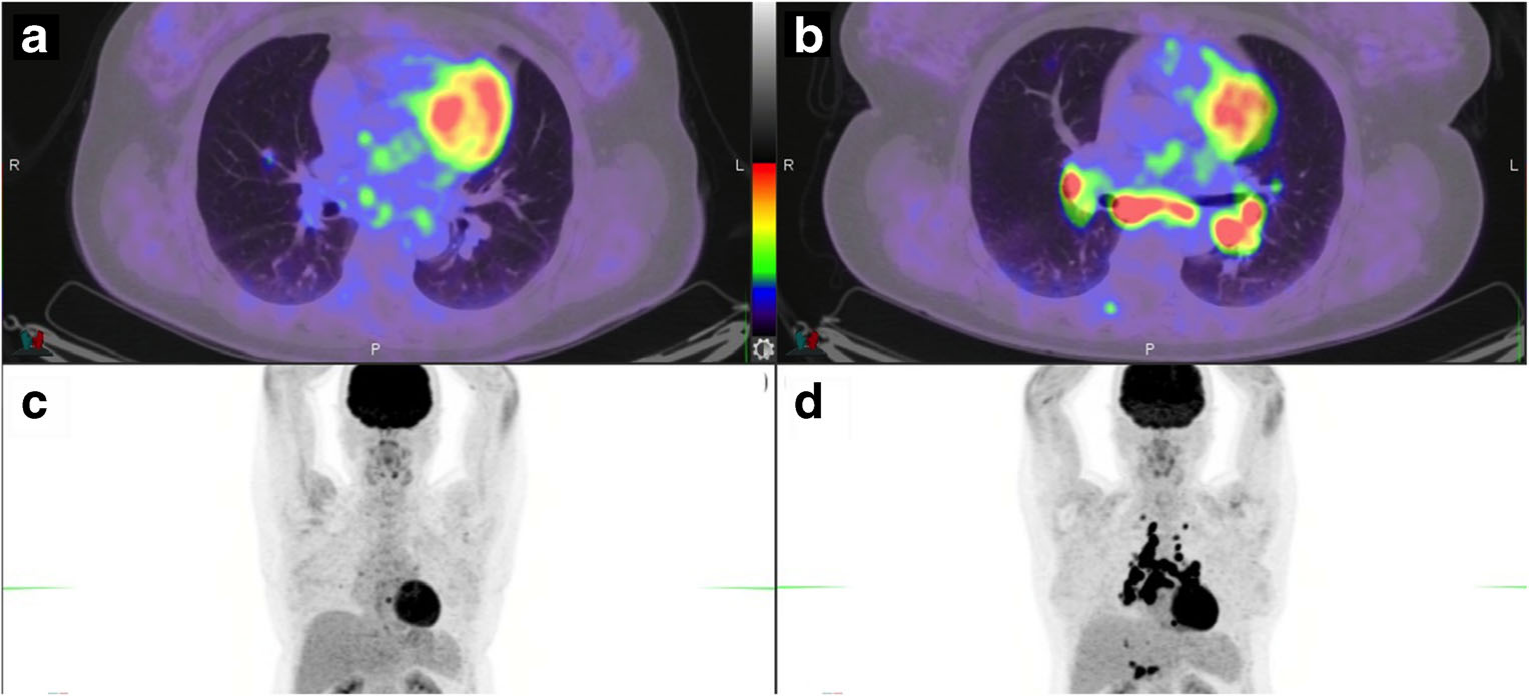
A patient with new pulmonary metastases (**a** fused PET/CT image, **c** maximum intensity projection image). Following treatment with pembrolizumab (**b** fused PET/CT image, **d** maximum intensity projection image), dramatic uptake is seen in symmetrical hilar, mediastinal and portocaval nodes indicating treatment-induced sarcoidosis. Prior small pulmonary nodules have resolved

Since every organ can be involved by the immune infiltrate, it is important to use the baseline scan data not only to compare changes in uptake in the target lesion(s) but also to check that intense uptake deemed to be an immune-related sign was not present on the baseline scan {for example colic and/or small-bowel uptake due to metformin, and diffuse thyroid uptake due to Hashimoto disease; Fig. 5). On the contrary, diffuse and intense uptake in these organs is likely to be an immune-related sign. One should also consider whether the pattern of new nodal uptake suggests sarcoidosis (lambda sign with or without portocaval nodes, Fig. 6).

### Therapy assessment

Depending on the availability of the SUV_peak_ metric on the workstation used, either the EORTC PET response criteria or PERCIST can be used to report FDG uptake changes in target lesion(s). However, because the patterns of response to immunotherapy are different from those to conventional chemotherapy and other molecularly targeted therapies, caution is required when reporting PET results in patients in whom disease progression is suspected, especially during the first few cycles of treatment. Attention should be paid to the possibility of pseudoprogression. In patients with apparent disease progression, the number and location of new lesions should be reported, excluding pathological foci in organs deemed to be due to the immune infiltrate. Indeed, a recent study suggested that the appearance of four or more new lesions of less than 1 cm in functional diameter or three or more new lesions of more than 1 cm in functional diameter is likely to be due to a real progression rather than pseudoprogression [24] (Table 2). Figures 1 and 2 show imaging in example patients with hyperprogression and pseudoprogression identified on FDG PET.

## Recommendations on data gathering

In addition to conventional SUV metrics, one could consider recording metabolic active tumour volume (MATV) and TLG before and after treatment, again excluding uptake in organs deemed to be due to the immune infiltrate. Indeed, MATV could be seen as the PET counterpart of iRECIST, where the sum of all lesions is used. More recently PET texture analysis [42] has emerged in the field of cancerology and has shown promising results in predicting response to treatment and patient survival. In addition to their potential role as prognosticators, the use of FDG PET heterogeneity parameters in differentiating between pseudoprogression and real progression could be evaluated, on the basis that pseudoprogressing lesions, due to the immune infiltrate, may have different heterogeneity patterns.

Similar to initiatives in the medical oncology community to gather large retrospective data to investigate new concepts such as hyperprogression [13, 14, 17], pooling data from different centres would allow a move forward in the identification of pseudoprogression using multiple PET quantitative parameters as described above. However, because MATV [43] and most textural features [44] are sensitive to PET reconstruction parameters, attention should be paid to the PET systems used before pooling data from different centres [45].
